# Biochemical, molecular, and in silico insights into the effects of cyphenothrin on *Culex quinquefasciatus* mosquitoes

**DOI:** 10.1186/s13071-026-07352-x

**Published:** 2026-03-13

**Authors:** Abhirup Saha, Subhajit Das, Prapti Das, Subhajit Ghosh, Abhinna Tamang, Subarna Thakur, Dhiraj Saha

**Affiliations:** 1 Insect Biochemistry and Molecular Biology Laboratory, Department of Zoology, University of North Bengal, Raja Rammohunpur, P.O. NBU, District: Darjeeling, Siliguri, West Bengal 734013 India; 2 Department of Bioinformatics, University of North Bengal, Raja Rammohunpur, P.O. NBU, Siliguri, 734013 India

**Keywords:** Cyphenothrin, *Culexquinquefasciatus*, Metabolic resistance, CYP450, Carboxylesterases, Pyrethroids

## Abstract

**Background:**

Cyphenothrin, a type II synthetic pyrethroid, is widely recommended by the World Health Organization (WHO) for control programs for mosquitoes such as *Culex quinquefasciatus*. This cosmopolitan mosquito species plays a pivotal role in transmitting several neglected tropical diseases, including lymphatic filariasis, West Nile virus, Japanese encephalitis, and so on. Besides treatment, vector control programs heavily rely on insecticides, leading to varying resistance due to prolonged exposure.

**Methods:**

The present study assessed the susceptibility status of field-collected *Cx. quinquefasciatus* larvae from two filarial endemic districts of sub-Himalayan West Bengal against cyphenothrin. All the wild populations have been exposed to their respective lethal concentration doses. Monooxygenase levels and carboxylesterase activities were quantified both in the wild and cyphenothrin-exposed populations. The expression profiles of selected carboxylesterase (*esterase A* and *esterase B*) and CYP450 (*CYP6AA7*, *CYP9J40,* and *CYP9J45*) genes were also assessed. Molecular docking analyses were performed to evaluate the binding affinities and interaction mechanisms of cyphenothrin with the selected proteins.

**Results:**

Most of the populations showed elevated resistance status to cyphenothrin. In enzymatic assays, elevated levels of monooxygenases and carboxylesterases are found both in the wild and cyphenothrin-exposed populations. The expression profiles of selected CYP450s and carboxylesterase genes indicated an upregulation in the studied wild and exposed populations. Furthermore, molecular docking simulations corroborated the strong binding affinities of cyphenothrin to these detoxification enzymes.

**Conclusions:**

These findings suggest the involvement of a few detoxifying genes in resistance development against cyphenothrin in *Cx. quinquefasciatus* mosquitoes and advocates the urgent need for regular resistance monitoring, molecular surveillance, gene knockdown studies, and incorporation of synergists into integrated vector management frameworks to sustain effectiveness of mosquito control programs.

**Graphical Abstract:**

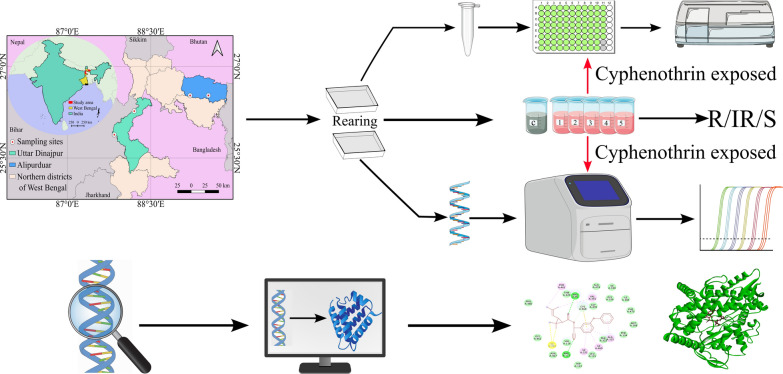

**Supplementary Information:**

The online version contains supplementary material available at 10.1186/s13071-026-07352-x.

## Background

Cyphenothrin (International Union of Pure and Applied Chemistry (IUPAC) name: [(*S*)-cyano-(3-phenoxyphenyl) methyl] (1*R*,3*R*)−2,2-dimethyl-3-(2-methylprop-1-enyl) cyclopropane-1-carboxylate) is a type II synthetic pyrethroid widely used as an insecticidal agent for controlling mosquitoes. They are chemical analogs of naturally synthesized pyrethrins, which can be derived from the flowers of *Chrysanthemum* spp. Similar to other pyrethroids, by affecting the voltage-gated sodium channels, it disrupts the nervous system of mosquitoes, leading to paralysis and eventual death. This class of insecticides is mostly recommended by the World Health Organization (WHO) for use in insecticide-treated nets and bed nets [1]. They are also used as space sprays and indoor residual sprays. Due to rapid actions and comparatively low mammalian toxicity of the recommended dose of cyphenothrin, it has been a preferred choice for mosquito control in many regions, including India [2]. However, long-term use of this insecticide may cause the development of resistance among mosquitoes.

Three major mosquito genera, namely *Aedes*, *Anopheles*, and *Culex*, are well known for transmitting a significant number of diseases and eventually posing public health risks. Among them, *Culex* mosquitoes are often more diverse and abundant than the other two genera [3]. This is because of their breeding habitat plasticity and adaptability to a wide range of environmental conditions [4]. Belonging to this genus, *Cx*. *quinquefasciatus*, or the southern house mosquito, is also widely distributed throughout the tropical and subtropical regions and transmits several neglected tropical diseases (NTD). Lymphatic filariasis is one of them, followed by West Nile fever, Rift Valley fever, sylvatic arboviruses, and so on. However, vaccines or medications for a few diseases such as West Nile fever are still under development or unavailable. Several outbreaks of this disease have been recorded since 1999, however, recently, Europe recorded 1200 West Nile Virus (WNV) cases, likely due to climate change and global warming [5]. Similar patterns of vector expansion may increase the risk of this disease in tropical and subtropical countries such as India. The emergence of WNV cases was recently recorded in states such as West Bengal, where the climate is mostly tropical [6]. As the *Cx. quinquefasciatus* is the most abundant mosquito species in the sub-Himalayan region of West Bengal, vector management is the only effective way to combat this disease [7].

Local authorities such as the National Center for Vector-Borne Disease Control (NCVBDC) recommend cyphenothrin (5% EC), along with other synthetic pyrethroids, as indoor residual sprays (IRS) and outdoor fogging for controlling this mosquito population [2]. However, widespread and repeated use of this insecticide causes resistance development against it, along with other synthetic pyrethroids, among *Cx. quinquefasciatus* populations of sub-Himalayan West Bengal [8, 9]. Over time, this selection pressure reduces the effectiveness of insecticides and makes vector control operations more difficult by allowing resistant individuals to flourish and proliferate. The mechanisms behind this resistance development were mostly correlated with target site resistance and metabolic resistance [8, 10, 11]. In the target site resistance, they change their insecticide binding sites so that insecticides cannot bind and work, whereas in the case of metabolic resistance through synthesizing more detoxifying proteins such as cytochrome P450 (CYP450), carboxylesterases, and glutathione S-transferase (GST), or by increasing these proteins’ efficacy, they detoxify insecticides at a faster rate [12]. Previous studies from this area have extensively explored both of these resistance mechanisms in wild *Cx. quinquefasciatus* populations, however, there remains a significant gap in understanding how metabolic enzyme activity, as well as their gene expression, varies after cyphenothrin treatment.

Cyphenothrin is primarily recommended for adult mosquito control through IRS and fogging [1, 2], however, resistance selection can occur across any life stages such as larvae due to repeated insecticide exposure in their habitats, including drains and stagnant water bodies. This exposure may come directly through the spraying and indirectly through rainfall runoff or improper disposal of residual insecticides. Larval-stage resistance surveillance is therefore a widely recognized, effective, and operationally feasible approach for early detection of resistance development and also for knowing underlying metabolic and molecular mechanisms. In the sub-Himalayan districts of West Bengal, routine resistance monitoring data, particularly at the larval stage of *Cx. quiquefasciatus*, are limited. The present study addresses this operational gap by generating a baseline larval susceptibility data against cyphenothrin and identifying biochemical and molecular mechanisms of resistance development that are relevant for optimizing both the larval and adult targeted control strategies.

CYP450 monooxygenases and CCEs were found to be associated with pyrethroid resistance in wild *Cx. quinquefasciatus* populations from this region previously [8, 9]. Concurrently, studies on the relative expression of a few selected detoxifying genes coding for these enzymes were also carried out from three districts, namely Darjeeling, Jalpaiguri, and Cooch-Behar of this sub-Himalayan West Bengal [9]. Therefore, this study investigated the status of wild as well as the dose-dependent effect of cyphenothrin on the monooxygenase level and carboxylesterase activity in the *Cx. quinquefasciatus* larvae from other two important filarial endemic districts of sub-Himalayan West Bengal, namely Alipurduar and Uttar Dinajpur. Simultaneously, expression levels of a few selected carboxylesterase and CYP450 genes were also studied before and after exposing cyphenothrin to different lethal concentrations in the larval stage of these populations. Molecular docking analyses were carried out to assess the binding affinities and potential interaction profiles of cyphenothrin with the selected detoxification-related proteins.

## Methods

### Study area

Two districts, namely Alipurduar and Uttar Dinajpur, were selected from the sub-Himalayan West Bengal on the basis of the vector abundance and endemicity of lymphatic filariasis. A previous study by Rudra and Mukhopadhayay [7] showed that *Cx. quinquefasciatus* is the most abundant mosquito species in this region. Both of the districts fall under the list of filarial endemic districts by the NCVBDC [13]. The districts were selected on the basis of the lymphatic filariasis endemicity instead of West Nile virus cases, due to the unavailability of district-wise reports for this disease. These districts are also considered significant in vector-borne disease dynamics due to their international borders. As Alipurduar shares a border with Bhutan and Uttar Dinajpur shares a border with Bangladesh. The climate of these districts is predominantly humid subtropical type (average annual temperature ranges from 23 °C to 28 °C) and experiences heavy to very heavy rainfall, especially during monsoon season. These environmental conditions are ideal for *Cx. quinquefasciatus* breeding and disease proliferation.

### Collection of mosquito larvae

**Fig. 1:**
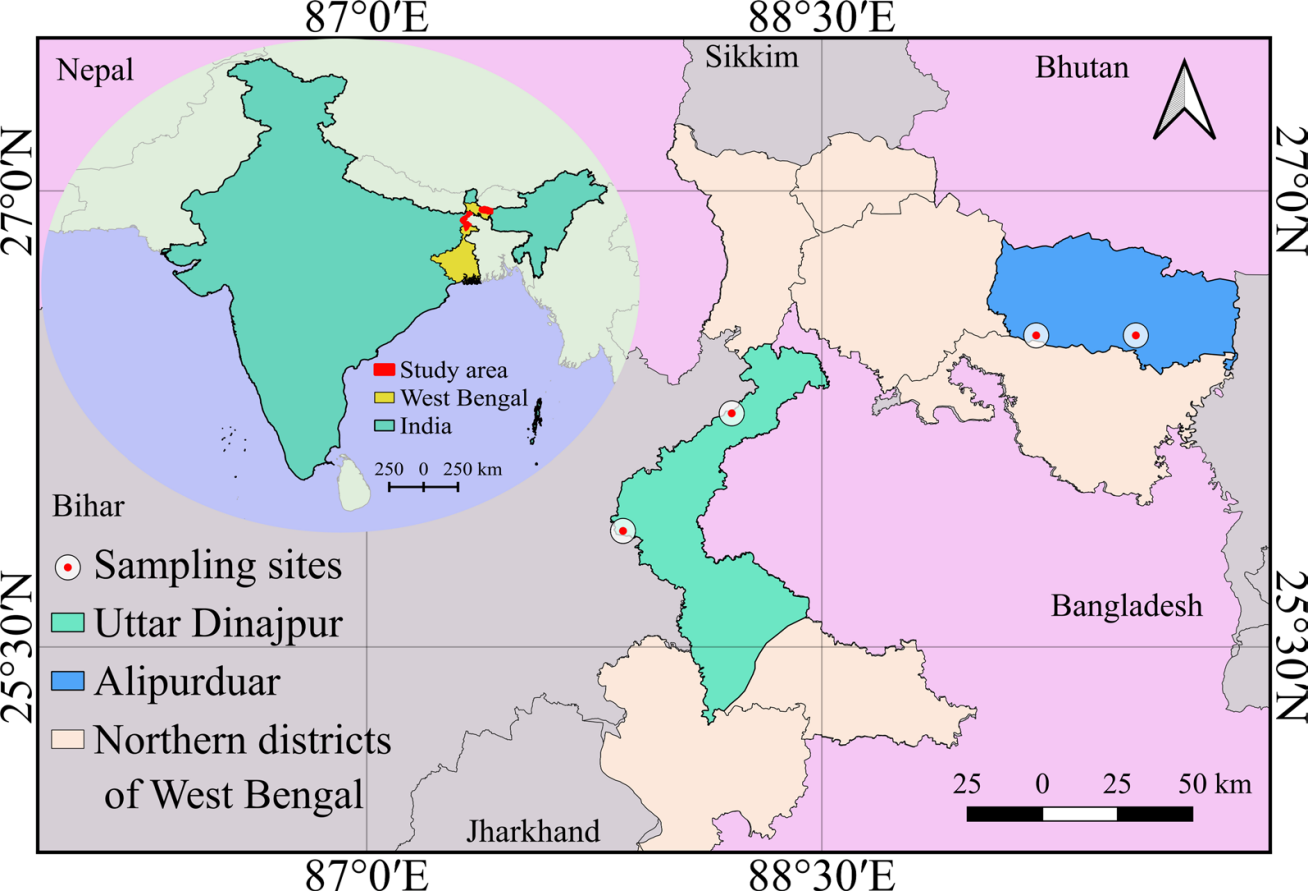
Sampling sites of *Cx. quinquefasciatus* across two districts (Alipurduar and Uttar Dinajpur) of sub-Himalayan West Bengal (map was prepared in QGIS 2025 [17], version 3.30.0)

Larvae of *Cx. quinquefasciatus* were collected from two districts of northern West Bengal (Fig. 1, Table 1). Two study sites from each district were selected during this study because of varying selection pressures and different resistance levels among mosquito populations. For example, Alipurduar (APD) and Falakata (FKT) from Alipurduar district, and Dalkhola (DKL) and Islampur (ISL) were selected from the Uttar Dinajpur district. For the rearing of the susceptible strain, mosquitoes were collected from the organically maintained botanical garden of the North Bengal University (NBU) campus. Sampling was done between February 2023 and January 2025 from different habitats such as drains, stagnant waters, and sewers from all the study sites twice a year. Each collected mosquito population was identified using different taxonomic keys [14–16] and reared for one generation (F1) to ensure population homogeneity for further experiments. With other species of insects including chironomids and drain flies, a few mosquito species such as *Armigeres* sp. and *Mansonia* sp. were also identified and separated during the study.

**Table 1:** Descriptions of the study sites with their geographical coordinates and co-existing species

District	Study area	Abbreviation used	Geographical coordinates	Coexistence of other species
Alipurduar	Alipurduar	APD	Lat 26.48°N Long 89.52°E	*Cx. pipiens*, chironomids
	Falakata	FKT	Lat 26.52°N Long 89.20°E	Chironomids, drain flies
Uttar Dinajpur	Dalkhola	DKL	Lat 25.89°N Long 87.82°E	Drain flies, *Mansonia* sp.
	Islampur	ISL	Lat 26.25°N Long 88.18°E	*Armigeres* sp., chironomids

### Susceptible population

Mosquito larvae were collected from the organically maintained medicinal garden of North Bengal University campus (26°42′34.03″ N; 88°21′14.96″ E). These larvae were reared for 30 generations, maintaining controlled physical parameters such as temperature (25° ± 2 °C) and relative humidity (70–80%). Mosquito rearing was done following standard protocol [18]. In the larval stage, they were given powdered fish feed, whereas in the case of adults, a 10% sucrose solution (Sigma, USA) soaked into pieces of cotton was given. A trimmed rat was provided as a blood meal for the female mosquitoes by obtaining approval from the Institutional Animal Ethics Committee (IAEC) (Reg no.: 840/G0/Re/S/04/CPCSEA), Department of Zoology, University of North Bengal.

### Larval bioassay and insecticide treatment

Larval bioassay was conducted using cyphenothrin (Merck, Germany), which is a recommended insecticide by NCVBDC for controlling *Cx. quinquefasciatus* mosquitoes [2]. In a previous study, the larval bioassay against cyphenothrin from these districts was conducted following the WHO protocols [31]. At that time, both lethal concentration 10 (LC_10_) and lethal concentration 50 (LC_50_) values were calculated from the same mortality datasets, but only the LC_50_ values were reported to analyze the resistance ratio 50 (RR_50_) values. For this study, the earlier data were combined with lethal concentration 10 (LC_10_) values. Both concentrations are important to assess sublethal effects on the biochemical changes of mosquito populations, as they show differential dose-dependent responses. Larval bioassay for the susceptible (SUS) population was conducted under the identical experimental conditions as those for the wild populations following the same WHO protocol [31], and the LC_50_ values were used for the calculation of RR_50_.

Serial dilution was performed from a 100 ppm (mg/L) stock solution of the cyphenothrin (Merck, Germany). Absolute ethanol was used as solvent. Firstly, concentrations were taken in a wide range to get 10–95% mortality. After that, lethal concentrations (LC_10_ and LC_50_) were calculated using a small range of concentrations dissolved in 100 ml of distilled water. Each beaker contained 20 larvae and had four replicates for each concentration, along with four control replicates containing an equivalent amount of ethanol and distilled water. After 24 h, mortality was recorded. Dead larvae were identified by probing the siphon with sharp needles; even the larvae exhibiting moribund conditions were also considered dead. Abbot’s formula was used when the mortality of the larvae in the control beaker falls between 5% and 20%, but more than 20% mortality among them is considered invalid. RR_50_ values were calculated following the WHO 2016 protocol (RR_50_ < 5: susceptible, RR_50_ 5–10: moderate resistance, and RR_50_ > 10: high resistance) [19].

$$\text{Corrected mortality}= \frac{{\% mortality in test}-{\%mortality in control}}{100-{\%mortality in control}} \times 100$$

$$RR_{50} =\frac{LC_{50}\text{ of the field population}}{LC_{50}\text { of the susceptible laboratory population}}$$

Groups of 100 early fourth instar *Cx. quinquefasciatus* larvae from each population were exposed to their sublethal concentrations (LC_10_ and LC_50_) of cyphenothrin. These concentrations were dissolved in 2000 ml of water. Three consecutive replicates were carried out for the whole setup. After 24 h of exposure, surviving larvae were selected for biochemical and gene expression studies.

### Biochemical assays

Biochemical assays were conducted on 90 larvae from each of the susceptible, wild, and cyphenothrin-treated mosquito populations following the WHO guideline [20]. For each assay, 30 larvae were pooled after being collected from different larval habitats within the respective study area to ensure biological representativeness. The sampling procedure was repeated three times and three independent pooled samples were generated to ensure biological replicates. Thus, from a single study site a total of 90 larvae were used for biochemical assay. 200 μl of distilled water was used to homogenize each mosquito larva, and the homogenization was carried out in a −20 °C minicooler. The homogenates were centrifuged at 1260 g for 1 min and then preserved at −20 °C. All the biochemical assays were carried out by taking supernatants from these preserved homogenates. Total protein content for each larva was calculated following the standard protocol [21], which was necessary to calculate other enzyme levels. Each of these assays was performed thrice using different mosquito larvae.

### Monooxygenase titration assay

Levels of monooxygenase were measured using 3,3’,5,5’-tetramethylbenzidine (TMBZ) and a 3% hydrogen peroxide solution following WHO protocol [20]. The endpoint assay was performed by reading the plates at 650 nm. This assay primarily measures the bound heme from the homogenates and approximately determines equivalent levels of cytochrome P450.

### Nonspecific esterase assay

Nonspecific esterase assays, including alpha and beta esterase assays, were carried out following the WHO protocol [20]. For alpha esterase activity, α-naphthyl acetate, and for beta esterase activity, β-naphthyl acetate, were used, and both of them were stained with Fast Blue BB salt stain solution. For the endpoint assay, the plates were read at 570 nm.

### Detoxifying gene expression analysis

The quantitative reverse transcriptase PCR (qRT-PCR) was used to assess the expression level of different CYP450 and carboxylesterase genes, selected on the basis of the literature review. These genes were found to be overexpressed in the pyrethroid resistant wild *Cx. quinquefasciatus* populations from sub-Himalayan West Bengal [9] and worldwide [22–24]. Cytochrome P450 genes, including *CYP6AA7*, *CYP9J40*, and *CYP9J45*, and carboxylesterase genes, including *esterase A* and *esterase B,* were chosen from previous literature [9]. The ribosomal protein L8 (*RPL8*) gene was used as a housekeeping gene to normalize the other detoxifying gene expressions. Table 2 comprises the list of primers used in the qRT-PCR. Three separate batches of mosquito larvae were used to replicate the entire process.

**Table 2:** List of primers used in quantitative PCR assays for gene expression analysis

Gene	Primers	Sequence (5’−3’)	Reference
*CYP6AA7*	F^a^	ATGACGCTGATTCCCGAGACTGTT	22
	R^b^	TTCATGGTCAAGGTCTCACCCGAA	
*CYP9J40*	F	ACCCGAATCCGGGCAAGTTTGAT	
	R	AACTCCAAACGGTAAATACGCCGC	
*CYP9J45*	F	TCAGCGGTACGGAAACGATGTGAT	23
	R	AGTCCATGTTGGTCTTCTGTCCCA	
*Esterase A*	F	GGCGCACTTGGTATGATATGTG	24
	R	TCTGGTCCTTTAGTCCGGCA	
*Esterase B*	F	ACGGTCCGGATTTC TTGGTT	
	R	TCCTGCACCGATTGACAACA	
*RPL8*	F	GCTGGCCGAAGGTGCGTGGT	
	R	TTGCGACCTGGCGGCGTTCC	

^a^F, forward; ^b^R, reverse

Total RNA was extracted by using Trizol reagent from early fourth instar larvae of susceptible, wild, and cyphenothrin-exposed wild *Cx. quinquefasciatus* populations from the study area. After checking purity using a spectrophotometer (SpectroSTAR Nano, BMG Labtech, Australia), cDNAs were prepared using a cDNA synthesis kit (Applied Biosystems, USA) and preserved at −20 °C. Quantitative real-time PCR (qRT-PCR) was performed using CFX 96 Real-Time PCR system (BIO-RAD, USA). Each reaction mixture contained 2 µl of cDNA (25 ng/μl), 10 µl of master mix (GoTaq^®^ qPCR Master Mix from Promega (USA)), forward and reverse primers at 10 µM concentration, and nuclease-free water, resulting in a final volume of 20 μl. These reactions and the negative control, which lacked the templates, were carried out in triplicate. The thermal cycler's conditions began with a 3-min initial denaturation or holding stage at 95 °C. Afterwards, there were 40 cycles of cycling at 95 °C for 3 s and 60 °C for 30 s. The melting curve analysis step was performed to verify the specificity of each reaction. The relative expression of these selected genes was calculated by the 2^−ΔΔCt^ method [25].

### In silico protein modeling and docking studies

Amino acid sequences of CYP6AA7 (GenBank ID: JF501089), CYP9J40 (GenBank ID: JF501091), CYP9J45 (GenBank ID: XM_001855163), and esterase A (GenBank ID: JQ812613) were retrieved from GenBank, and the sequence of esterase B (Vectorbase ID: CPIJ013917) was retrieved from VectorBase. The three-dimensional structures of these proteins were predicted using AlphaFold (version 3.0) [26]. Predicted local distance difference test (pLDDT) scores [27] were also predicted for model selection, and the model with the highest scores was selected for further analysis. The predicted models were validated using the PROCHECK web server [28, 29].

The protein models and ligands were then prepared with the OPLS3 force field using Schrödinger (Maestro Version 12.5.139, Schrödinger, LLC, New York, NY). The chemical structure of cyphenothrin (PubChem CID: 38,283) was retrieved in SDF (Structure-Data File) format from the National Center for Biotechnology (NCBI) PubChem database (https://pubchem.ncbi.nlm.nih.gov/). The probable binding sites of each protein were predicted using the P2RANK web server [30]. Receptor grids for each protein were generated using Glide (Maestro Version 12.5.139, Schrödinger, LLC, New York, NY) on the basis of the predicted ligand-binding coordinates. The binding affinities of cyphenothrin with each protein were recorded using Glide’s Extra Precision (XP) docking mode. Docked complexes were visualized using BIOVIA Discovery Studio Visualizer.

### Statistical analysis

For all the statistical analysis, SPSS software (version 21) was used. On the basis of concentration/mortality data, probit analysis was carried out to determine both the LC_10_ and LC_50_ values. One-way analysis of variance (ANOVA) and independent samples *t*-tests were carried out to examine the variation of enzyme levels of monooxygenases or activities of carboxylesterases among susceptible, wild, and cyphenothrin-exposed wild populations. Similar tests were also carried out to examine differential expression levels of the selected genes among these populations.

## Results

### Larval bioassay

By analyzing the RR_50_ values, it was observed that except for APD, all the studied populations, namely ISL, DKL, and FKT, showed high resistance against cyphenothrin, as their RR_50_ values were greater than ten (Table 3), whereas APD was moderately resistant (RR_50_ = 10.00). Extreme resistance was found in the DKL population, with maximum RR_50_ (RR_50_ = 37.45) as well as LC_50_ value (LC_50_ = 1.071 mg/L; 95% CI 0.833–1.430 mg/L) from all the other studied populations. The LC_50_ values were not correlated with LC_10_ values, as the doses varied across different studied populations. For instance, in the case of the DKL population, the highest LC_50_ value was observed (LC_50_ = 1.071 mg/L; 95% CI 0.833–1.430 mg/L), whereas its LC_10_ value (LC_10_ = 0.225 mg/L; 95% CI 0.118–0.335 mg/L) was lower than a few other studied populations such as ISL (LC_10_ = 0.236 mg/L; 95% CI 0.116–0.351 mg/L) and FKT (LC_10_ = 0.275 mg/L; 95% CI 0.158–0.372 mg/L).

**Table 3:** Larval bioassay results showing the response of different wild *Cx. quinquefasciatus* populations to cyphenothrin

Population	LC_10_^a^(mg/L) (95% CI^c^)	LC_50_^b^(mg/L) (95% CI)	Slope ± SE^d^	*r*^2 e^	RR_50_^f^
ISL	0.236 (0.116–0.351)	0.766 (0.567–1.017)	2.22 ± 0.201	0.964	26.78
DKL	0.225 (0.118–0.335)	1.071 (0.833–1.430)	1.96 ± 0.078	0.968	37.45
APD	0.082 (0.051–0.113)	0.286 (0.230–0.348)	2.23 ± 0.023	0.969	10.00
FKT	0.275 (0.158–0.372)	0.644 (0.505–0.823)	3.13 ± 0.280	0.981	22.52
SUS	0.011 (0.005–0.016)	0.0286 (0.0207–0.0372)	2.64 ± 0.49	0.982	–

^a^*LC*_10_ lethal concentration 10, ^b^*LC*_50_ lethal concentration 50, ^c^*CI* confidence interval; ^d^*SE* standard error, ^e^*r*^2^ regression coefficient; ^f^*RR*_50_ resistance ratio at lethal concentration 50

### Monooxygenase titration assay

**Fig. 2:**
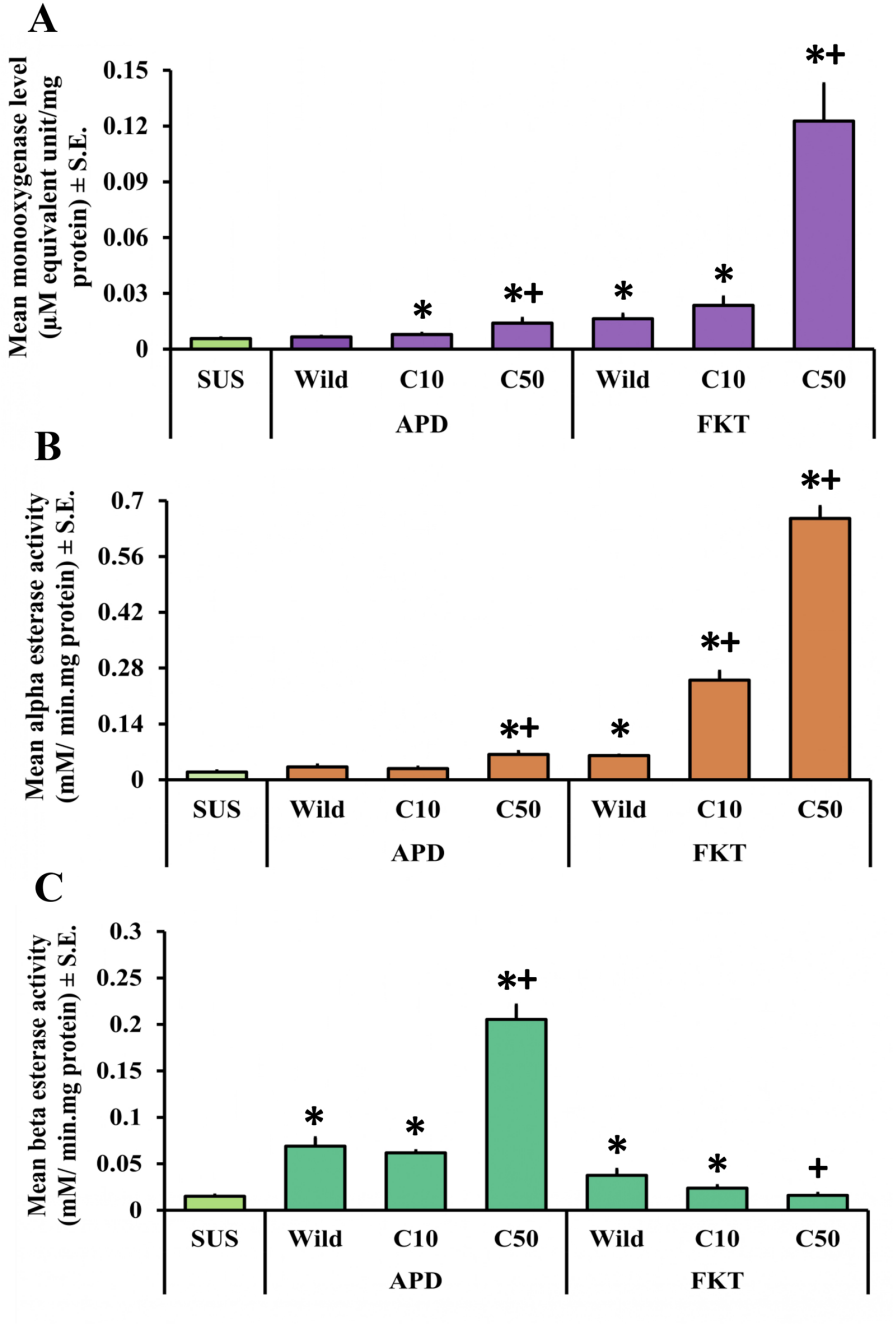
Graph showing comparative levels of monooxygenase (**A**) and the activities of alpha esterases (**B**) and beta esterases (**C**) (mean ± SE) in wild and cyphenothrin-exposed larvae of *Cx. quinquefasciatus* mosquitoes from APD and FKT populations. Bars with “ * ” depict significantly different from SUS population (*P* < 0.05) and bars with “ + ” depict significantly different from wild population (*P* < 0.05). C10 and C50 indicate cyphenothrin-exposed populations at LC_10_ and LC_50_ concentrations, respectively

**Fig. 3:**
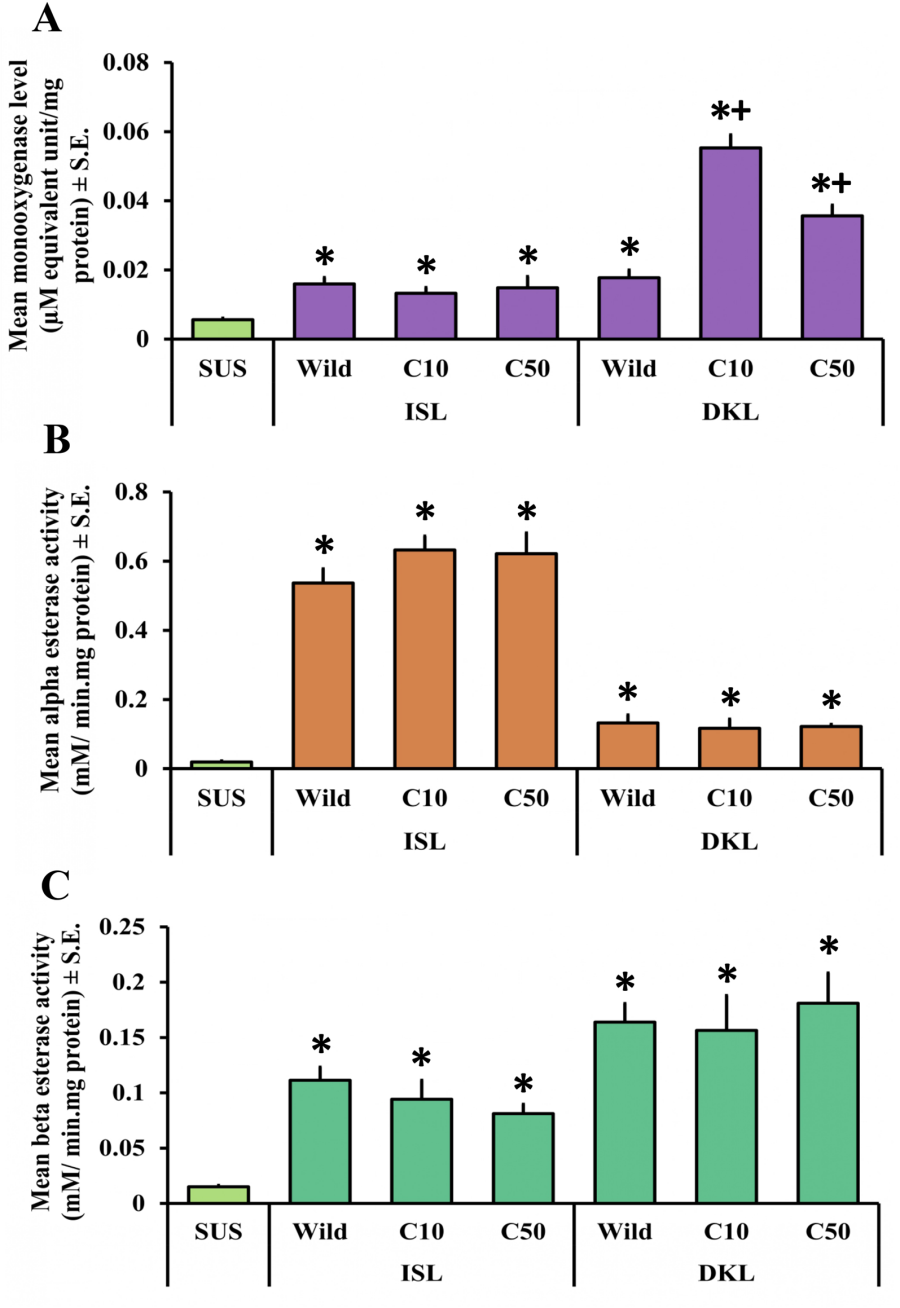
Graph showing comparative levels of monooxygenase (**A**) and the activities of alpha esterase (**B**) and beta esterases (**C**) (mean ± SE) in wild and cyphenothrin exposed larvae of *Cx. quinquefasciatus* mosquitoes from ISL and DKL populations. Bars with “ * ” depict significantly different from SUS population (*P* < 0.05) and bars with “ + ” depict significantly different from wild population (*P* < 0.05). C10 and C50 indicate cyphenothrin-exposed populations at LC_10_ and LC_50_ concentrations, respectively

Elevated monooxygenase levels were observed in both the wild and treated populations (Fig. 2A and Fig. 3A). Except for APD, all other wild populations, namely ISL, DKL, and FKT, showed significantly higher levels of monooxygenase than that of the SUS population (*P* < 0.05). Among them, the maximum level of monooxygenase was observed in the DKL, followed by FKT and ISL. All three of these populations showed an approximately threefold higher monooxygenase level than the laboratory-reared SUS population (*P* < 0.05). After giving the exposure with cyphenothrin in their respective lethal doses (LC_10_ and LC_50_), most of the population showed increased level of monooxygenase; however, significant increases from the wild population were mostly recorded after giving the exposure with LC_50_ values. Interestingly, in the ISL population, levels of monooxygenase were decreased slightly from the wild after the treatment. Consequently, in the DKL population in both of the lethal doses, monooxygenase levels were significantly higher than the wild populations; however, maximum levels were recorded after exposure with the LC_10_ dose. In the cases of APD and FKT, monooxygenase levels showed a gradual surge through increasing cyphenothrin concentrations. The highest monooxygenase level was observed in the FKT population at the LC_50_ exposure, which was approximately 7.5-fold higher (*P* < 0.05) than that of the wild population.

### Nonspecific esterase assay

Elevated level of both the carboxylesterases (alpha and beta) was detected in all of the wild and cyphenothrin-treated populations (Fig. 2B, C and Fig. 3B, C). Among the wild populations, apart from APD, which showed nonsignificant but increased alpha esterase activity, all others showed significantly elevated alpha and beta esterase activities compared with the SUS population (*P* < 0.05). Maximum alpha esterase activity within the wild populations was reported from the ISL, whereas maximum beta esterase activity was reported from the DKL population. Following the treatment with cyphenothrin, the majority of the populations showed higher alpha esterase activity than their wild populations. However, in the DKL population, the alpha esterase activity decreased slightly after exposure. A significant (*P* < 0.05) increase in the alpha esterase activity compared with the wild population after LC_50_ exposure was observed in the APD and FKT populations.

In addition, beta esterase activity after cyphenothrin exposure was differential but mostly decreased from the wild in most of the populations, such as ISL and FKT, for both the LC_10_ and LC_50_ doses, and for APD and DKL at the LC_10_ dose. In the case of FKT, after giving the exposure with the LC_50_ concentration, beta esterase activity significantly decreased from the wild population. Even for all the populations, after giving exposure to their respective LC_10_ doses, beta esterase activities were decreased from their wild values. However, after exposure to the LC_50_ values of DKL and APD, beta esterase activity was increased; even in the case of APD, it was significantly (*P* < 0.05) increased, with nearly threefold higher activity than that of the wild population.

### Detoxifying gene expression analysis

**Fig. 4:**
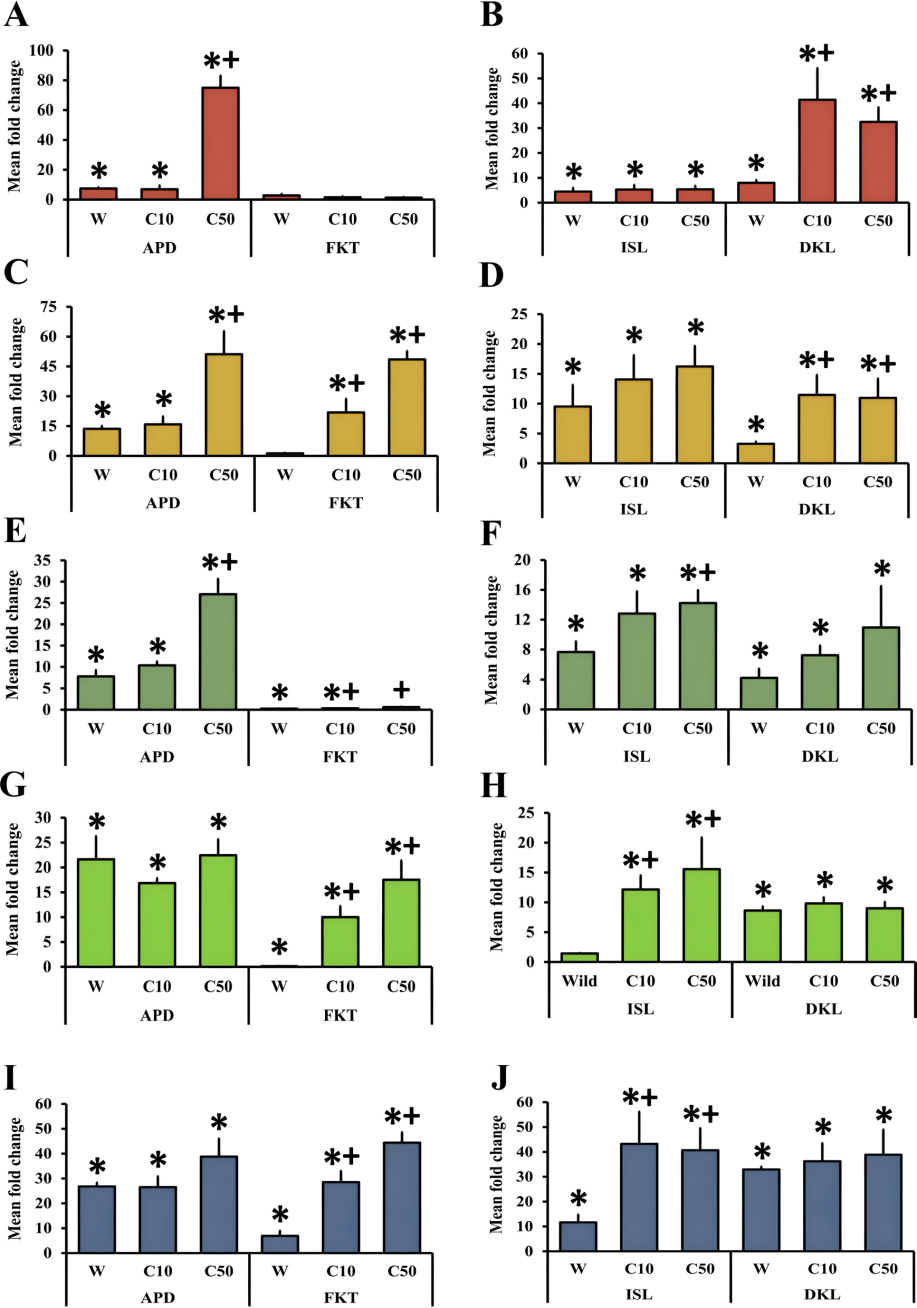
The relative expression levels (mean ± SE) of selected detoxifying genes among the wild and cyphenothrin exposed *Cx. quinquefasciatus* populations from the study area. **A**, **B**: *CYP6AA7*; **C**, **D**: *CYP9J40*; **E**, **F**: *CYP9J45*; **G**, **H**: *esterase A*; and **I**, **J**: *esterase B*. Bars with “ * ” were significantly different from SUS population (*P* < 0.05) and bars with “ + ” were significantly different from wild population. C10 and C50 indicate cyphenothrin-exposed populations at LC_10_ and LC_50_ concentrations, respectively

Among all the selected genes, a differential expression pattern was observed throughout the wild as well as exposed populations (Fig. 4). In the case of carboxylesterase genes, *esterase B* was more overexpressed than *esterase A* in the wild. All four populations had shown significant (*P* < 0.05) overexpression of *esterase B,* whereas in the case of *esterase A,* only DKL and APD had shown significant overexpression (*P* < 0.05) than that of the laboratory-reared SUS population. Maximum relative expression for the *esterase A* was reported from the APD population, and maximum relative expression for the *esterase B* was recorded from the DKL population. Further, all the selected CYP genes (*CYP9J45*, *CYP6AA7*, and *CYP9J40*) were found to be significantly overexpressed (*P* < 0.05) among most of the studied wild populations, except FKT. Relative overexpression (relative expression > 2) for *CYP6AA7* was also recorded from the FKT population, but not significant (*P* < 0.05) from the SUS.

After exposure to cyphenothrin, the surviving larvae have mostly shown increased relative expression than their wild populations in terms of all the selected CYP and carboxylesterase genes. However, the expression pattern was differentiating population to population and in different doses. In the case of ISL, both the esterase genes have been increased from fourfold to tenfold, respectively, than their wild expression, whereas in the case of CYP genes, *CYP6AA7* was more or less similar, but *CYP9J45* and *CYP9J40* have been increased ~twofold than their wild population expression. A similar type of results was also recorded from the FKT population, where *esterase A*, which was underexpressed in the wild population, increased approximately 9–17-fold than that of the SUS population upon cyphenothrin exposure. Simultaneously, *esterase B* increased approximately fourfold to sixfold upon exposure to different doses of cyphenothrin from their wild population. Among the CYP genes of FKT, only *CYP9J40* has shown 17–38 times overexpression than the wild, whereas the other two CYP genes were almost the same or little decreased from the wild population. On the contrary, in the case of DKL, both the esterase genes were nearly similar among the wild and exposed populations, but CYP genes increased compared with their wild populations, such as *CYP6AA7* around fivefold, *CYP9J45* around twofold, and *CYP9J40* around threefold. At the same time, in the case of APD, *esterase A* was almost the same or little decreased after exposure to cyphenothrin, whereas *esterase B* was slightly increased (about 1.5 fold) after exposure to the LC_50_ dose of cyphenothrin compared with their wild populations. In all the populations, most of the relative gene expression was increased from LC_10_ to LC_50_ (*P* < 0.05). However, in a few cases, such as DKL for *esterase A*, ISL for *esterase B*, DKL and FKT for *CYP6AA7*, and DKL for *CYP9J40,* relative gene expression was similar or slightly lower in the LC_50_ exposed populations than that of the LC_10_.

### Protein structure modeling and docking

The predicted protein structures obtained from AlphaFold revealed that CYP6AA7 consists of 509 amino acids, CYP9J40 524 amino acids, and CYP9J45 542 amino acids, while esterase A and B consist of 540 amino acids. Validation through PROCHECK analysis revealed that more than 90% of the amino acid residues in all proteins were located in favorable regions, except for esterase B. Esterase B had 89.5% of residues in favorable regions, however, it does not have any residues in the disallowed regions. These results indicate that all predicted models are reliable for docking analysis (Supplementary file 1).

**Fig. 5:**
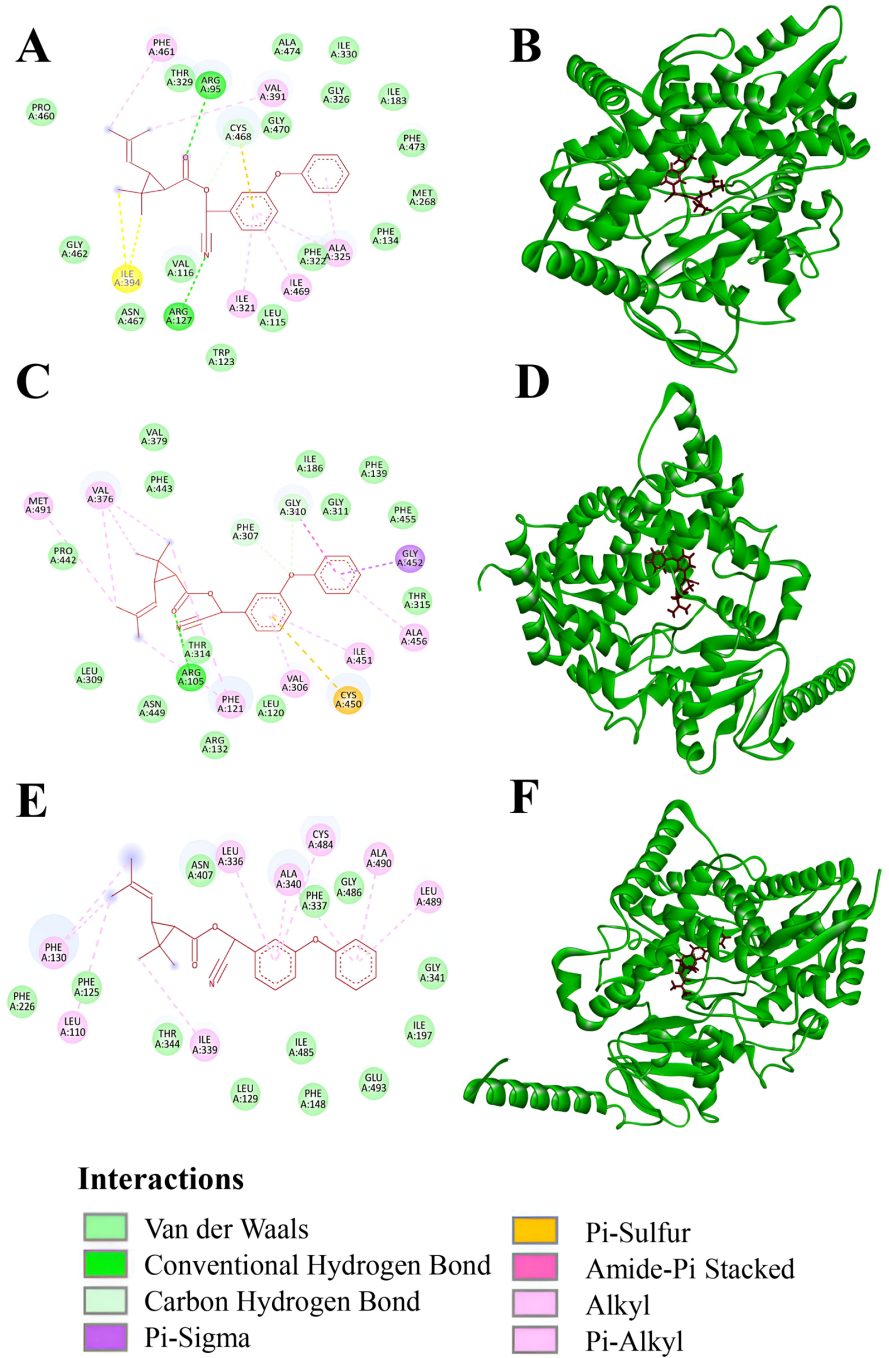
Molecular docking of selected CYP proteins with cyphenothrin. **A** Two-dimensional (2D) interaction of CYP9J40 with cyphenothrin highlighting key binding residues. **B** Three-dimensional (3D) interaction of CYP9J40 (green) with cyphenothrin (red). **C** 2D interaction of CYP6AA7 with cyphenothrin highlighting key binding residues. **D** 3D interaction of CYP6AA7 (green) with cyphenothrin (red). **E** 2D interaction of CYP9J45 with cyphenothrin highlighting key binding residues. **F** 3D interaction of CYP9J45 (green) with cyphenothrin (red)

**Fig. 6:**
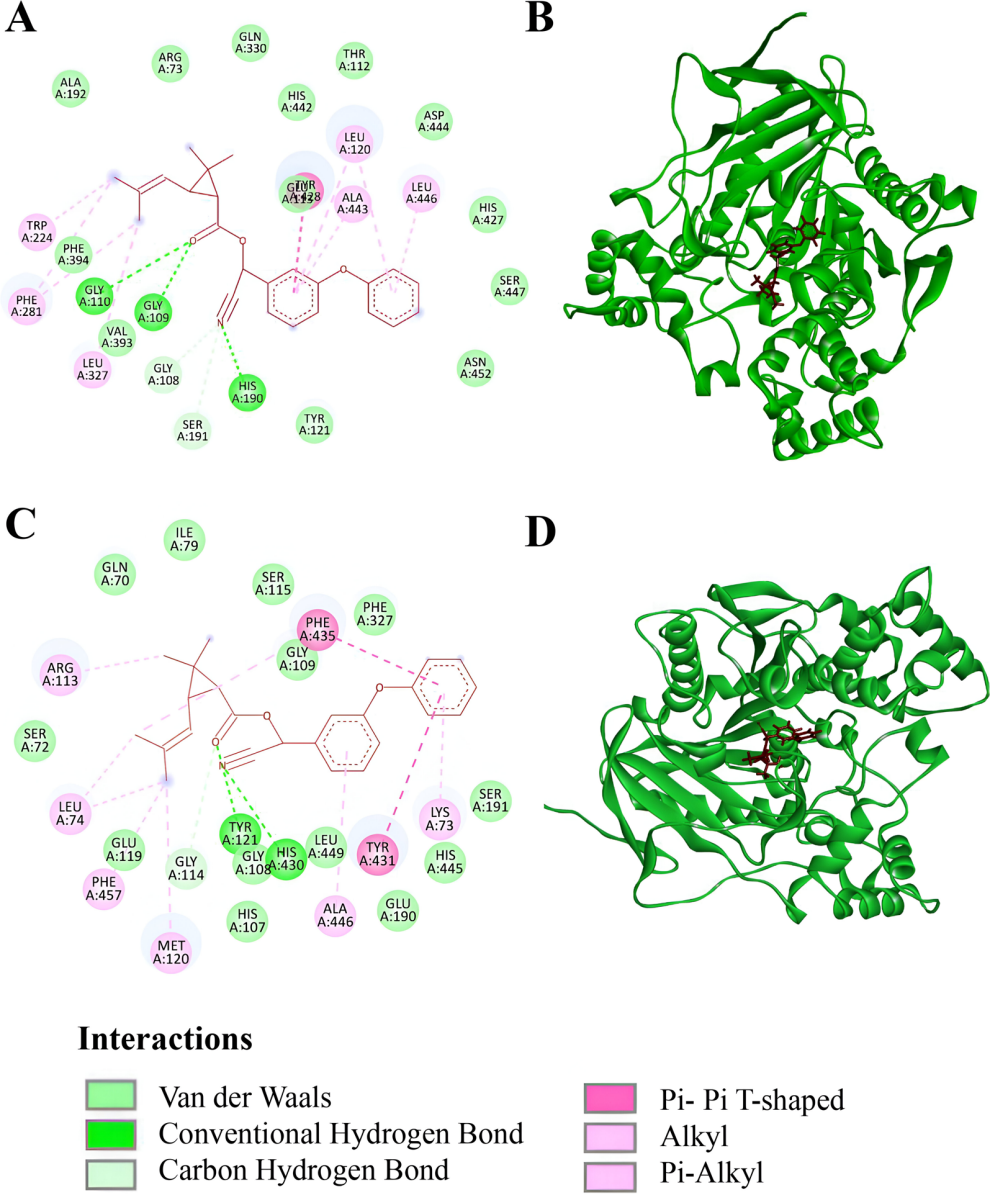
Molecular docking of selected carboxylesterase proteins with cyphenothrin. **A** 2D interaction of esterase B with cyphenothrin highlighting key binding residues. **B** 3D interaction of esterase B (green) with cyphenothrin (red). **C** 2D interaction of esterase A with cyphenothrin highlighting key binding residues. **D** 3D interaction of esterase A (green) with cyphenothrin (red)

Molecular docking of cyphenothrin with the selected detoxification enzymes (CYP6AA7, CYP9J40, CYP9J45, esterase A, and esterase B) revealed a range binding scores (Table 4 and Fig. 5 & 6). Esterase B and cyphenothrin complex recorded the highest binding score (−9.511 kcal/mol), followed by CYP9J40 (−8.367 kcal/mol), CYP6AA7 (−7.611 kcal/mol), and CYP9J45 (−7.526 kcal/mol). Weakest binding was recorded in interaction with esterase A (−5.593 kcal/mol). These cyphenotrin and enzyme interactions were stabilized by combination of both polar and hydrophobic association, such as hydrogen bonds, Pi-alkyl interactions, and so on. Among all the CYP proteins, in the case of CYP9J40 the highest binding affinity was observed, with the maximum number of conventional hydrogen bonds formed with ARG127 and ARG95, followed by π-sulfur interactions with ILE394 and CYS468 residues. Additionally, several alkyl and π-alkyl interactions were also detected. Simultaneously among the carboxylesterase genes, esterase B revealed the strongest binding interactions, characterized by multiple conventional hydrogen bonds (GLY109, GLY110, HIS190) followed by other hydrophobic interactions, including carbon hydrogen bonds (GLY108, SER19), π–π T-shaped interaction (TYR428), and several alkyl and π-alkyl interactions.

**Table 4:** Molecular docking analysis results of the selected genes with cyphenothrin

Protein	UniProt ID	Docking score (kcal/mol)	Notable interactions
CYP6AA7	G3F973	−7.611	Conventional H_2_ Bond: ARG105 π-Sigma: GLY452 π-Sulfur: CYS450 Amide-π stacked: GLY310 Carbon hydrogen bond: PHE307, GLY310 Alkyl/π-alkyl: ALA456, ILE451, VAL306, PHE121, MET491, VAL376
CYP9J40	G3F974	−8.367	Conventional H_2_ Bond: ARG127, ARG95 π-Sulfur: ILE394, CYS468 Alkyl/π-alkyl: PHE461, VAL391, ALA325, ILE469, ILE321
CYP9J45	A0A1Q3FIK5	−7.526	Alkyl/π-alkyl: ILE339, LEU110, PHE130, LEU336, ALA340, CYS484, ALA490, LEU489
Esterase A	A5Y5K2	−5.593	Conventional H_2_ Bond: TYR121, HIS430 Carbon hydrogen bond: GLY114 π–π T-shaped: PHE435, TYR431 Alkyl/π-alkyl: LYS73, ALA446, MET120, PHE457, LEU74, ARG113, GLY109
Esterase B	B0X3V9	−9.511	Conventional H_2_ Bond: GLY109, GLY110, HIS190 Carbon hydrogen bond: GLY108, SER191 π–π T-shaped: TYR428 Alkyl/π-alkyl: TRP224, PHE281, LEU327, LEU120, ALA443, LEU446

## Discussions

The burden of *Culex*-borne diseases is increasing in northern West Bengal, as the emergence of WNV cases and most of the districts are categorized as filariasis endemic by NCVBDC [6, 13]. The trend is anticipated to grow along with ongoing population growth. In addition, vector control has now become difficult due to the development of resistance among them. Since the discovery of resistance between different mosquito vectors in the 1950 s, global research has deeply focused on understanding the underlying mechanisms of insecticide resistance. Revealing these mechanisms is crucial for the development of novel strategies and techniques for effective insecticide use. The current study provides new insights into the development of biochemical resistance among *Cx. quinquefasciatus* larvae against a type II synthetic pyrethroid, cyphenothrin, which is recommended by NCVBDC. Alongside the current work, it offers the first insight into differential exposure of cyphenothrin on the CYPs and carboxylesterases in the larval stages of wild *Cx. quinquefasciatus* populations from two filariasis-endemic districts of sub-Himalayan West Bengal, India.

Previous studies from these districts have shown diverse patterns of resistance against cyphenothrin among *Cx. quinquefasciatus* populations [31]. Similar results have also been found in this study. Except for APD, all of the studied population were completely resistant to cyphenothrin, which may be due to the frequent use of synthetic pyrethroids in domestic mosquitocidal items such as liquid vaporizers, repellent coils, space sprays, thermal fogging, indoor residual sprays (IRS), and so on [32]. As a type II synthetic pyrethroid, cyphenothrin has extensive applications in both public health and agricultural fields [2]. The highest cyphenothrin resistance was observed in the DKL population, followed by ISL and FKT in this study. This can be attributed to their nature as rapidly developing urbanized cities; as a result, frequent insecticide exposure occurs through the household, spraying, intensive agricultural practices, etc. Similar types of higher insecticide resistance against cyphenothrin were also recorded among the urban strains of *Aedes aegypti* from Malaysia [33]. Another study by Zarog and Elssaidi also showed notably high resistance levels to cyphenothrin in the *Cx. pipiens* populations [34]. In contrast, APD showed incipient resistance, probably because it was closer to forested, ecologically less disturbed areas with minimal pesticide application in agriculture and vector management than the other selected populations. Due to lower selection pressure, APD was found to be incipiently resistant; however, it can eventually develop high resistance in the future, as its RR_50_ value was ten, if proper management is not executed. The findings also showed that not all the populations showed a consistent correlation between LC_10_ and LC_50_ values. In certain populations, the LC_50_ values were quite high, and the corresponding LC_10_ values were surprisingly lower than those in other populations. This suggests the possibility of variation in the slope of the dose–response curve, indicating differing patterns of susceptibility. These results also depict the importance of different sublethal concentrations and their dose–response patterns. Previously, resistance to other synthetic pyrethroids, such as permethrin, deltamethrin, and lambda-cyhalothrin, has also been reported in *Cx. quinquefasciatus* populations from these two districts. High resistance to cyphenothrin from the studied area can also be correlated with the development of cross-resistance among them due to the extensive application of different synthetic pyrethroids. A study conducted by Weerasinghe et al. (2001) [35] also demonstrated that permethrin-resistant *Cx. quinquefasciatus* can exhibit cross-resistance to cyphenothrin. Moreover, a high level of pyrethroid resistance has been reported among various *Culex* populations worldwide [36]. Therefore, regular monitoring is necessary to detect and prevent resistance and cross-resistance in the studied region.

Both the treated and untreated wild *Cx. quinquefasciatus* larvae from all the studied populations had shown elevated levels of monooxygenases and increased carboxylesterase activity than that of the laboratory-reared SUS population. This suggests the role of metabolic resistance against cyphenothrin-like type-II synthetic pyrethroids. Even previous studies by Rai et al. [8, 10] and Saha et al. [9] have also demonstrated the role of these detoxifying enzymes in the establishment of resistance against different pyrethroid insecticides among *Cx. quinquefasciatus* populations from this region. There have also been reports of elevated monooxygenase levels and carboxylesterase activities in pyrethroid-resistant *Cx. quinquefasciatus* populations from different parts of the world, including Mexico [37], Florida [38], and Malaysia [39], among others. It was clear from combining the findings of the monooxygenase titration assay and the larval bioassay that the larval *Cx. quinquefasciatus* populations from the wild exhibiting greater levels of resistance to cyphenothrin also had higher levels of monooxygenases. For example, DKL had the highest resistance levels, as its RR_50_ value was the maximum among the four, and it also showed the highest monooxygenase level. Simultaneously, APD exhibited a moderate resistance status (RR_50_ 5–10), with the lowest RR_50_ value, and also showed the lowest monooxygenase levels. Although the ISL population exhibited a slightly higher resistance ratio (RR_50_) than the FKT, the monooxygenase levels were almost equal, and the FKT had slightly higher monooxygenase levels than ISL. However, the relatively greater resistance observed in the ISL population compared with the FKT could be a reason for the elevated alpha esterase activity, which was the highest among all the tested populations. Even the beta esterase activity was higher in ISL than that of the FKT population. Moreover, among the studied wild populations, DKL has shown comparatively higher levels of the detoxifying enzymes, whereas APD has shown just the opposite, which can be correlated with their resistance status against cyphenothrin. Both of these enzymes are responsible for the degradation of cyphenothrin-like type-II synthetic pyrethroids [40].

The findings also indicate that exposure to cyphenothrin causes a gradual increase in terms of both the CYP levels and carboxylesterase activities among most of the studied populations. This can be a result of the induction effect of cyphenothrin on these detoxifying enzyme classes. These elevated enzyme levels help in faster detoxification of insecticides. After exposure, beta esterase activities were more or less similar or slightly higher than those of their wild populations. It has been previously reported that alpha esterase activity plays a more significant role in the development of pyrethroid resistance compared with beta esterase activity in different *Culex* species [41]. Altogether, both of their significantly higher activities than those of the laboratory-reared SUS population confirm the link between these two carboxylesterases in resistance development against cyphenothrin. Interestingly, in a few studied populations, such as CYP levels in the ISL population, alpha esterase activities in the DKL population, and beta esterase activities in the ISL, DKL, and FKT, populations were roughly similar before and after exposure to cyphenothrin. This is likely due to prior exposure to pyrethroids in the environment or due to other resistance mechanisms, as the emergence of pyrethroid resistance is a multifaceted process that may encompass various resistance mechanisms concurrently. The degree of enzyme induction was also varied with the different concentrations of cyphenothrin. The majority of the studied populations showed higher levels of monooxygenases and higher carboxylesterase activities in LC_50_ surviving larvae compared with those that survived LC_10_ exposure, indicating an enzyme-mediated dose-dependent adaptive response to cyphenothrin stress. This suggests that mosquitoes from the studied populations that survive higher concentrations of cyphenothrin may have developed more metabolic resistance. However, a few exceptions were also observed, where surviving LC_10_ larvae showed comparatively higher enzyme levels or activities, possibly because of early induction of detoxifying enzymes or inherent variability of enzymes within those populations. This varied response to cyphenothrin exposure may be the consequence of differential changes at the transcriptional or translational levels of these detoxifying enzymes [40]. This could indicate that metabolic resistance has evolved independently in each mosquito population over time. Another study by Das et al. [42] also found differential levels of monooxygenases in *Ae. albopictus* populations upon exposure to deltamethrin from this area. For a better understanding of the population-specific variations in response to different pyrethroids, further studies focused on how alterations are occurring in these enzyme levels across multiple mosquito populations belonging to the same species are required.

To find potential candidate genes associated with cyphenothrin resistance, the expression levels of three CYP genes and two carboxylesterase genes were examined using qRT-PCR analysis in the wild and cyphenothrin-treated *Cx. quinquefasciatus* populations. Among these carboxylesterase genes, *esterase B* and all the studied CYP genes were found to be significantly overexpressed among most of the studied wild populations. Overexpression of these genes among wild *Cx. quinquefasciatus* populations were also found in the adjacent districts, such as Darjeeling, Jalpaiguri, and Coochbehar [9]. Several studies have also reported the role of these detoxifying genes in pyrethroid-resistant *Cx. quinquefasciatus* populations. A study confirms overexpression of *CYP6AA7* along with other CYP genes, such as *CYP6Z2* and *CYP9J34*, in the field-collected permethrin-resistant *Cx. quinquefasciatus* populations from Tamil Nadu, India [43]. Similarly, in our study, *CYP6AA7* and *CYP9J40* were found to be significantly overexpressed among the three study sites, while *CYP9J45* was overexpressed in two study sites, demonstrating the roles of these detoxifying genes in the development of cyphenothrin resistance. Not only CYP genes, but also carboxylesterase genes, were found to be overexpressed among insecticide-resistant mosquito populations. According to a recent study conducted by Talipouo et al. [24], the overexpression of *esterase A* and *esterase B*, along with other CYP genes, was found to be responsible for enhancing resistance against bendiocarb and malathion insecticides. Simultaneously, in this study, *esterase B* was found to be significantly overexpressed among all the sampling sites, whereas overexpression of *esterase A* was found among two sites, though their roles in cyphenothrin resistance require functional validation.

Cyphenothrin exposure resulted in up-regulation of the selected genes among most of the studied populations, depicting its induction effect on CYP and carboxylesterase enzymes. Liu et al. [22] reported similar findings, indicating the overexpression of four CYP genes after exposure to permethrin in resistant *Cx. quinquefasciatus* larvae. Simultaneously, *CYP6AA7* and *CYP9M10* were found to be overexpressed in the permethrin-selected *Cx. quinquefasciatus* population [44]. Carboxylesterases are also considered to play a major role in pyrethroid resistance through the up-regulation of their genes in several insects [45]. Previous studies have reported overexpression of carboxylesterase genes in pyrethroid-resistant *Cx. quinquefasciatus* populations, which may also explain the higher expression of *esterase A* and *esterase B* genes upon exposure to cyphenothrin in this study [43]. The present study also found that cyphenothrin exposure led to both up- and downregulation of the same gene in different study areas. For instance, *CYP6AA7* was upregulated in ISL and DKL but downregulated in the FKT population. Simultaneously, in the case of APD, it was first downregulated upon exposure to the lower dose (LC_10_); however, significantly (*P* < 0.05) upregulated than that of the wild population upon exposure to the higher dose (LC_50_) of cyphenothrin. These variations in responses among different studied populations and under varying insecticide doses point toward the potential for independent evolution of resistance. Similar results have also been reported by Das et al. [42] in *Ae*. *albopictus* populations from the adjacent areas. These studies emphasize the importance of conducting population-specific studies on different mosquito species to better understand the metabolic resistance patterns, a key factor in vector control strategies.

Cyphenothrin and cyp/cce interaction analysis also reveal differential involvement of various cce and cyp genes, which corroborates the observed expression pattern. Esterase B, with comparatively higher docking score, suggests that cyphenothrin fits snugly into the active site of esterase B, which is stabilized by hydrogen bond and other hydrophobic interactions. Efficient binding promotes hydrolyzation of cyphenothrin by nucleophilic attack. Carboxylesterase is primarily linked with organophosphate metabolism but pyrethroid metabolism by carboxylesterase is not unlikely, as mentioned in the previous paragraphs. Docking analysis by Gong et al. [46] also suggests that three novel carboxylesterases have the potential to metabolize permethrin in *Cx. quinquefasciatus* mosquitoes, which ultimately contributes to metabolic resistance. A similar observation made by Li et al. [47] demonstrated that two carboxylesterase genes can effectively metabolize beta-cypermethrin in *Plutella xylostella*. In another study, Feng and Liu [48] validate that carboxylesterase can metabolize permethrin in the housefly (*Musca domestica*) through functional analyses. These results strengthen the hypothesis that metabolic resistance via carboxylesterases contributes significantly to pyrethroid detoxification in the studied mosquito populations.

Another isoform, esterase A, showed less effective binding with cyphenothrin. Low binding score indicate that this isoform of esterase may bind with pyrethroid only for their sequestration. This result highlights isoform-specific differences in substrate recognition—not all esterase will contribute equally to detoxification.

Cytochrome P450 monooxygenases demonstrated significant interaction with cyphenothrin, with CYP9J40 exhibiting the most robust binding stabilization through multiple hydrogen bonds and hydrophobic interactions. This interaction may facilitate the effective oxidation of cyphenothrin. CYP enzymes metabolize pyrethroids, converting them into more polar hydroxy-pyrethroids via hydroxylation, thereby promoting their excretion. Docking results suggest that this process is primarily mediated by CYP9J40. Other CYP proteins, such as CYP6AA7 and CYP9J45, which have comparatively lower binding scores, may not directly interact with cyphenothrin but instead sequester the alcohol moiety produced by carboxylesterase for further excretion. The presence of hydrophobic amino acids, including isoleucine, phenylalanine, and valine, near the cyphenothrin binding sites may enhance the hydrophobic binding of insecticides, facilitating the subsequent hydroxylation of cyphenothrin, a critical step in the detoxification of pyrethroids [49]. These results indicate the direct and indirect involvement of these cce and cyp proteins to reduce the effect of cyphenothrin and development of pyrethroid resistance.

Previously, pyrethroid resistance was linked to CYP450 monoxygenases and carboxylesterases in the wild *Cx. quinquefasciatus* from the adjacent area by using inhibitors such as piperonyl butoxide (PBO) and triphenyl phosphate (TPP) [10]. However, this study provides additional evidence that corroborates the contribution of these detoxifying enzymes through the overexpression of multiple genes. In addition, for the first time, this study reported the role of these detoxifying genes in developing resistance against type II synthetic pyrethroid cyphenothrin.

## Conclusions

The present findings highlight that the differential regulation of detoxifying enzymes, especially monooxygenase and carboxylesterases, plays a crucial role in the emergence of cyphenothrin resistance in *Cx. quinquefasciatus* mosquitoes. The multifactorial processes of resistance development, as observed by the heterogeneity in enzyme expression among populations, is likely shaped by the local selection pressures, insecticide usage patterns, and genetic background. To understand the fate of cyphenothrin after entering resistant *Cx. quinquefasciatus* mosquitoes, more gene knockdown studies are needed, which can help to understand resistance development. The results of this study emphasize the urgent need for continuous resistance monitoring, molecular surveillance, and incorporation of synergists into integrated vector management frameworks to sustain the effectiveness of *Cx. quinquefasciatus* control programs.

## Supplementary Information

Additional file 1.

## Data Availability

Data supporting the main conclusions of this study are included in the manuscript.

## Abbreviations

NTD: Neglected tropical disease
NCVBDC: National Center for Vector Borne Diseases Control
IAEC: Institutional Animal Ethics Committee
WHO: World Health Organization
GST: Glutathione S-transferase
APD: Alipurduar
FKT: Falakata
ISL: Islampur
DKL: Dalkhola
LC_10_: Lethal concentration 10
LC_50_: Lethal concentration 50
RR_50_: Resistance ratio 50
PBO: Piperonyl butoxide
TPP: Triphenyl phosphate
