# Supplementary material for: Biochemical, molecular, and in silico insights into the effects of cyphenothrin on Culex quinquefasciatus mosquitoes
Source: Parasit Vectors. 2026 Mar 13;19:175. doi: 10.1186/s13071-026-07352-x (PMC13097794; doi:10.1186/s13071-026-07352-x)
Supplement: Supplementary file 1 — Additional file 1. [file 13071_2026_7352_MOESM1_ESM.pdf]

1. CYP6AA7

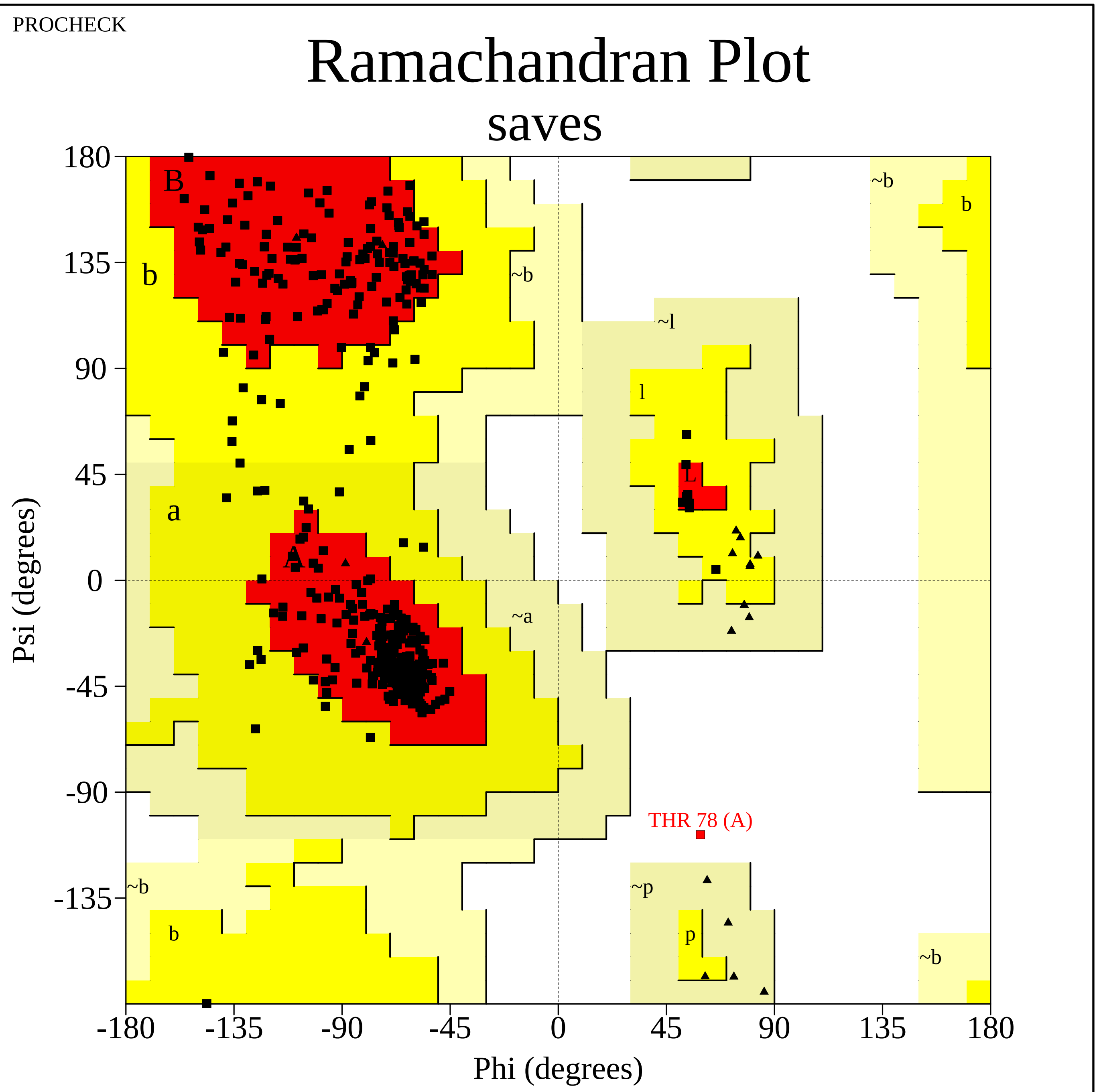

Plot statistics

Residues in most favoured regions [A,B,L]	416	91.6%
Residues in additional allowed regions [a,b,l,p]	37	8.1%
Residues in generously allowed regions [~a,~b,~l,~p]	0	0.0%
Residues in disallowed regions	1	0.2%
	----	------
Number of non-glycine and non-proline residues	454	100.0%
Number of end-residues (excl. Gly and Pro)	2	
Number of glycine residues (shown as triangles)	25	
Number of proline residues	28	

Total number of residues	509	

Based on an analysis of 118 structures of resolution of at least 2.0 Angstroms and R-factor no greater than 20%, a good quality model would be expected to have over 90% in the most favoured regions.

2. CYP9J40

PROCHECK

Ramachandran Plot

saves

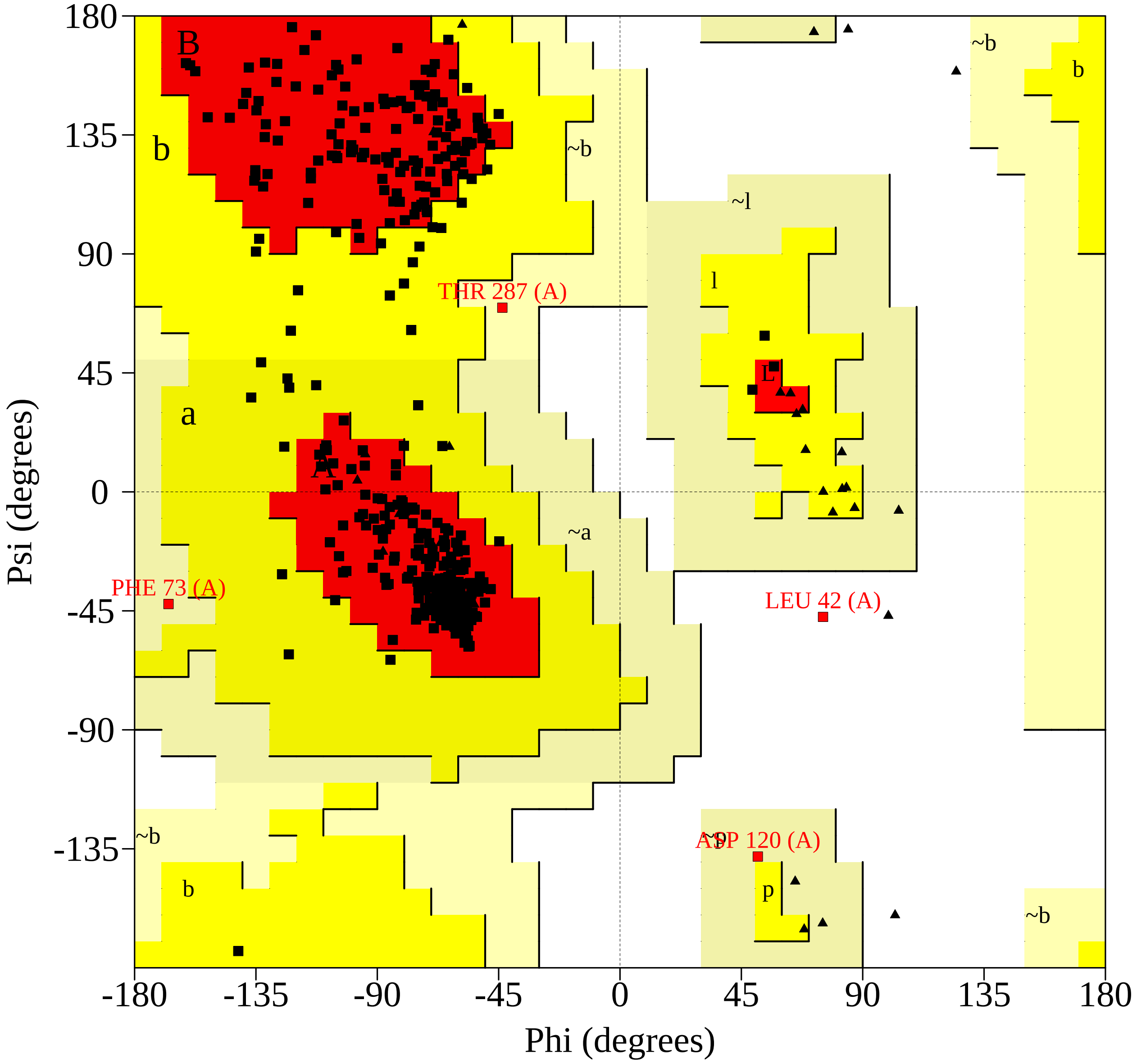

Plot statistics

Residues in most favoured regions [A,B,L]	429	92.7%
Residues in additional allowed regions [a,b,l,p]	30	6.5%
Residues in generously allowed regions [~a,~b,~l,~p]	3	0.6%
Residues in disallowed regions	1	0.2%
	----	------
Number of non-glycine and non-proline residues	463	100.0%
Number of end-residues (excl. Gly and Pro)	2	
Number of glycine residues (shown as triangles)	37	
Number of proline residues	22	

Total number of residues	524	

Based on an analysis of 118 structures of resolution of at least 2.0 Angstroms and R-factor no greater than 20%, a good quality model would be expected to have over 90% in the most favoured regions.

saves_01.ps

3. CYP9J45

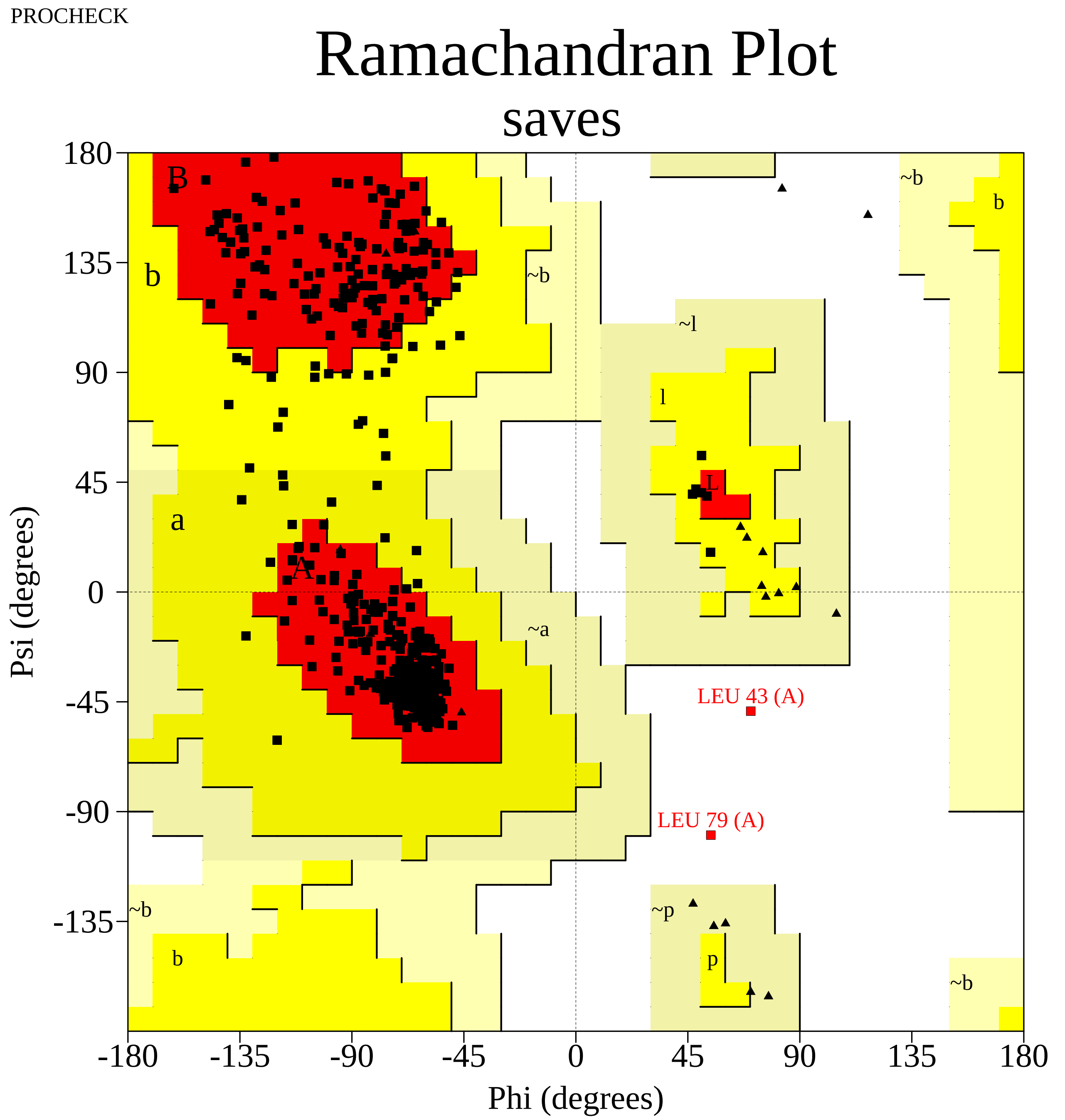

Plot statistics

Residues in most favoured regions [A,B,L]	445	91.4%
Residues in additional allowed regions [a,b,l,p]	40	8.2%
Residues in generously allowed regions [~a,~b,~l,~p]	0	0.0%
Residues in disallowed regions	2	0.4%
	----	------
Number of non-glycine and non-proline residues	487	100.0%
Number of end-residues (excl. Gly and Pro)	2	
Number of glycine residues (shown as triangles)	30	
Number of proline residues	23	

Total number of residues	542	

Based on an analysis of 118 structures of resolution of at least 2.0 Angstroms and R-factor no greater than 20%, a good quality model would be expected to have over 90% in the most favoured regions.

saves_01.ps

4. esterase A

PROCHECK

Ramachandran Plot

saves

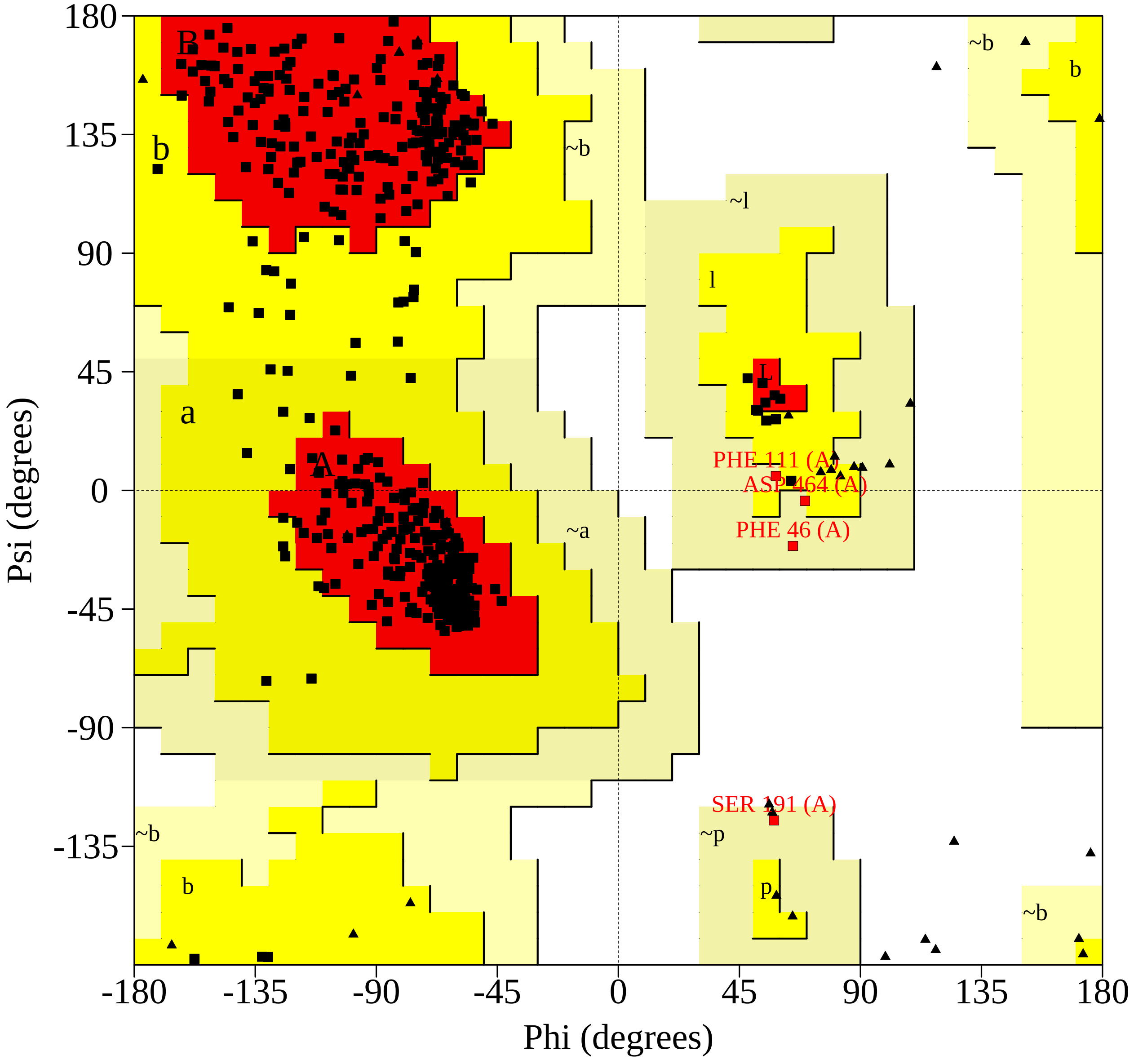

Plot statistics

Residues in most favoured regions [A,B,L]	418	90.5%
Residues in additional allowed regions [a,b,l,p]	40	8.7%
Residues in generously allowed regions [~a,~b,~l,~p]	4	0.9%
Residues in disallowed regions	0	0.0%
	----	------
Number of non-glycine and non-proline residues	462	100.0%
Number of end-residues (excl. Gly and Pro)	2	
Number of glycine residues (shown as triangles)	44	
Number of proline residues	32	

Total number of residues	540	

Based on an analysis of 118 structures of resolution of at least 2.0 Angstroms and R-factor no greater than 20%, a good quality model would be expected to have over 90% in the most favoured regions.

5. esterase B

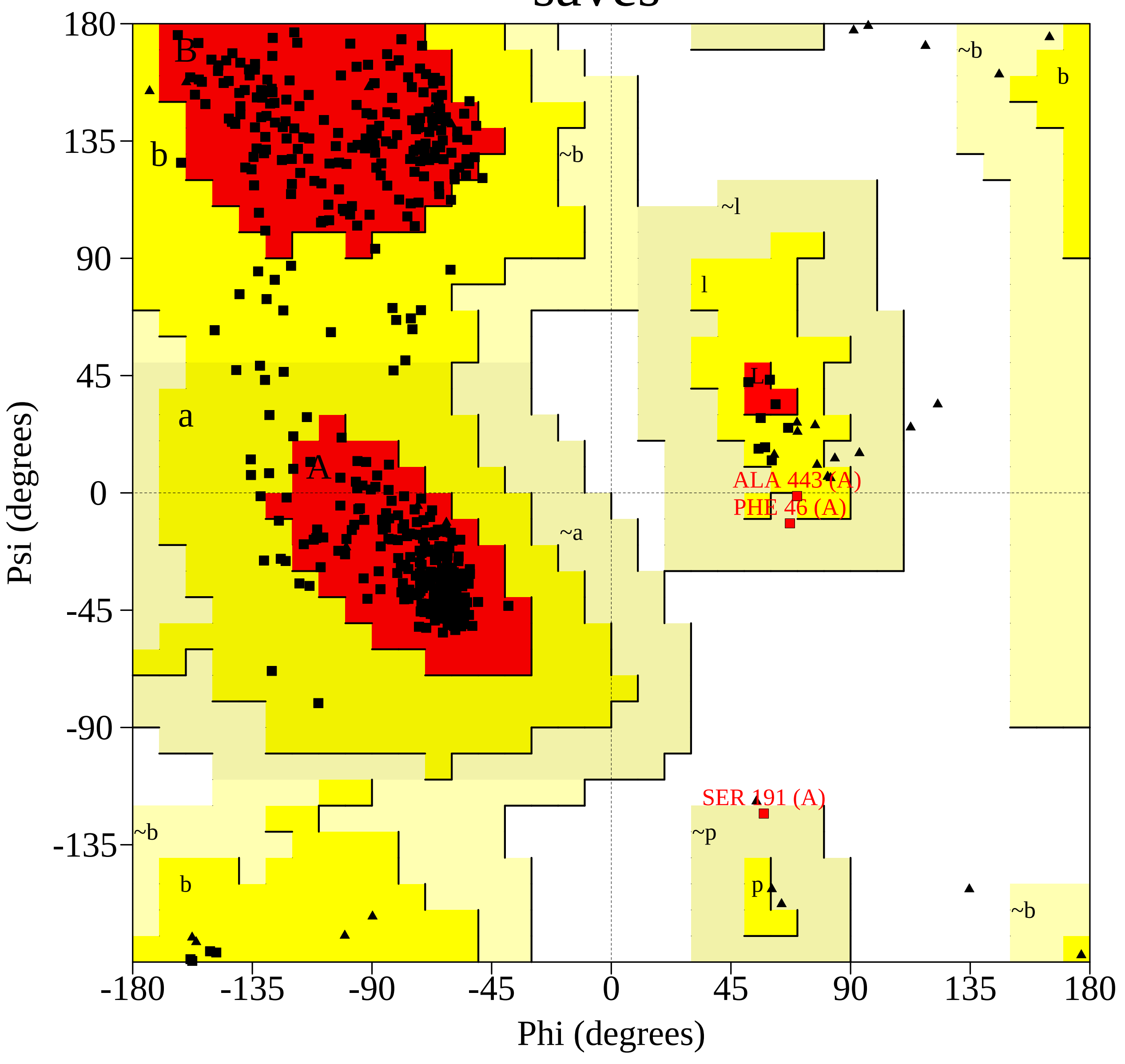

Plot statistics

Residues in most favoured regions [A,B,L]	416	89.5%
Residues in additional allowed regions [a,b,l,p]	46	9.9%
Residues in generously allowed regions [~a,~b,~l,~p]	3	0.6%
Residues in disallowed regions	0	0.0%
	----	------
Number of non-glycine and non-proline residues	465	100.0%
Number of end-residues (excl. Gly and Pro)	2	
Number of glycine residues (shown as triangles)	42	
Number of proline residues	31	

Total number of residues	540	

Based on an analysis of 118 structures of resolution of at least 2.0 Angstroms and R-factor no greater than 20%, a good quality model would be expected to have over 90% in the most favoured regions.

saves_01.ps
